# Relationship Between Sural Nerve Function, Physical Function, and the Ability to Perform Activities of Daily Living in Hospitalized Elderly Patients With Proximal Femoral Fractures: A Prospective Observational Study

**DOI:** 10.7759/cureus.80596

**Published:** 2025-03-14

**Authors:** Eisuke Takeshima, Akira Kimura

**Affiliations:** 1 Department of Physical Therapy, Faculty of Health and Medical Sciences, Hokuriku University, Kanazawa, JPN; 2 Department of Health Science, Graduate School of Health Sciences, Gunma Paz University, Takasaki, JPN

**Keywords:** elderly patient, functional independence measure, peripheral neuropathy, proximal femoral fractures, sural nerve function

## Abstract

Background

Falls in older adults, specifically causing proximal femoral fractures, greatly affect daily functioning and life expectancy. Peripheral neuropathy is an established risk factor for falls. However, the relationship between nerve conduction velocity (CV), action potential (AP), and fall risk or functional recovery after rehabilitation is not well understood. This study aimed to clarify whether sural nerve function can predict fall risk and functional improvement in activities of daily living.

Methods

We assessed elderly patients hospitalized with proximal femoral fractures in a convalescent rehabilitation ward. Sural nerve function was evaluated using the DPNCheck (NeuroMetrix, Inc., Woburn, MA, USA), with additional data collected on demographics, blood tests, medication use at admission and discharge, incidence of falls during hospitalization, physical function (Short Physical Performance Battery, or SPPB), and activities of daily living (Functional Independence Measure, or FIM). Statistical analyses explored the associations between the sural nerve CV/AP and the collected measures.

Results

In 27 patients (7 men and 20 women), sural nerve CV and AP were significantly correlated with both SPPB and FIM scores. Logistic regression identified FIM toileting as a significant predictor of reduced sural nerve AP (<5 μV; odds ratio: 3.22, p = 0.025). Multiple regression analysis showed that age and AP were significant predictors of the total FIM cognitive scores at discharge, with an adjusted R² of 0.464.

Conclusion

This study suggests that sural nerve function may help predict functional abilities in patients with proximal femoral fractures, particularly in the FIM motor tasks. Assessing peripheral nerve function can enhance rehabilitation plans by targeting fall risk management and promoting improvements in daily activities.

## Introduction

The number of falls among the elderly is predicted to increase as the global population ages [1,2]. Falls in the elderly can lead to decreased physical function and mobility and are a serious health concern that can shorten life expectancy [3]. Internal factors related to physical function and mobility, as well as external environmental factors, such as the type of flooring and footwear [4], interact in complex ways to influence fall risk. Although exercise-based interventions have been shown to be effective in preventing falls in community-dwelling older adults [5], no consensus exists on fall prevention for hospitalized and institutionalized older adults [6,7]. This lack of consensus may be because of the high-risk nature of these populations, who are more prone to falls owing to multiple illnesses and frailty. Therefore, interventions effective for healthier, community-dwelling older adults may not be effective for high-risk groups that are prone to falls. It is necessary to assess the risk of falls and consider effective interventions devised for this group.

The timed up-and-go test, one-leg standing time test, functional reach test, Berg balance scale, and postural sway test are all well-known methods for assessing fall risk [8,9]. However, these assessment methods are for individuals who can move independently. Elderly individuals in hospitals and care facilities, who often face acute and chronic illnesses, represent a high-risk group that is challenging to assess with these methods. Therefore, current assessment practices may be inadequate.

Peripheral neuropathy, associated with falls in elderly individuals, has received increasing attention in recent years. Diabetes mellitus is a common disease also associated with falls and fractures [10,11]. Cancer chemotherapy [12], metabolic syndrome [13], and certain nutrients (calcium and magnesium [14], vitamin B6 [15], and dietary phosphorus [16]) are associated with peripheral neuropathy. Additionally, age-related peripheral nerve degeneration occurs independently [17]. Previous studies have shown that peripheral neuropathy is an important risk factor for falls [18,19]. However, the relationship between peripheral nerve conduction velocity (CV) and action potentials (APs) is related to physical function and mobility. Elucidation of this aspect may play an important role in predicting fall risk in older adults. The DPNCheck (NeuroMetrix, Inc., Woburn, MA, USA) device used in this study is a point-of-care testing (POCT) tool that can rapidly and quantitatively assess CV and AP and is expected to provide a new perspective on fall risk assessment and help identify elderly patients at risk of falling.

Against this background, the aim of this study was to evaluate the peripheral nerve function of elderly patients who had suffered a proximal femoral fracture during their hospital stay using the DPNCheck and to clarify how CV and AP are related to physical function and the ability to perform activities of daily living. In addition, we have investigated whether the assessment of peripheral nerve function is useful in determining the risk level of groups at high risk of falling, which is difficult to determine using conventional assessment methods.

## Materials and methods

This study included elderly patients with proximal femoral fractures who were admitted to the convalescent rehabilitation ward of Tsurugi Hospital between June 2023 and March 2024. Approval was obtained from the Tsurugi Hospital Ethics Committee (No. 5-2) and the Gunma Paz University Graduate School of Medicine Ethics Committee. Informed consent was obtained from participants following both verbal explanation and written consent. The study was conducted in accordance with the tenets of the Declaration of Helsinki. This study was registered with the University Hospital Medical Information Network (UMIN000054233).

Patient selection

The inclusion criteria were patients aged 60 years or older who sustained a proximal femoral fracture between June 2023 and March 2024, underwent surgery at Tsurugi Hospital or another hospital, and were admitted to the convalescent rehabilitation ward of Tsurugi Hospital. Exclusion criteria included age under 60 years and significant communication difficulties (especially those with severe dementia, aphasia, or severe hearing or visual impairment).

Assessment of falls during hospitalization

A fall is defined by the WHO as "an event in which a person unintentionally comes to rest on the ground, floor, or other low surface" [20]. The presence or absence of falls was confirmed using electronic medical records and observation notes by medical staff. If a patient experienced at least one fall during hospitalization, it was classified as "fall present."

Assessment of medication, physical function, and mobility

Information on the types, dosages, and effects of medications taken by the patients during their hospital stay was collected from electronic medical records.

Physical function was assessed using the Short Physical Performance Battery (SPPB) at both admission and at discharge. The SPPB consists of three tests: balance, walking, and sit-to-stand. Each test was scored out of 4, and a total score of 12 was used to determine the results. The results were graded as follows: 0-6 points indicate low performance, 7-9 points indicate standard performance, and 10-12 points indicate high performance [21].

Muscle strength in knee extension was assessed using the Daniels and Worthingham Muscle Test (MMT) evaluation method at the time of admission and discharge [22]. The measurement method was as follows [23]:

Test Position

The patient sat upright with the knee fully extended at 0°, avoiding knee hyperextension. Verbal instructions were given: "Straighten your knee." The hand-giving resistance was contoured on top of the leg, just proximal to the ankle. The other hand was positioned under the thigh and above the knee. The examiner then stated, "Hold it. Do not let me bend it," and assigned a score of Grades 3, 4, or 5.

If Weaker Than Grade 3

The patient lay on the non-testing side, with the examiner standing behind the patient at knee level. For stability, the untested leg was flexed if needed. One arm cradled the leg around the thigh, with the hand supporting the underside of the knee. The other hand held the leg just above the ankle. The examiner stated, "Straighten your knee." Grade 2 was assigned if the patient could extend the knee.

If Weaker Than Grade 2

The patient was positioned supine, and the examiner stated, "Push the back of your knee down" or "Tighten your knee cap," while palpating the quadriceps tendon. Grades of 1 or 0 were assigned based on the response.

The ability to perform activities of daily living was assessed using the Functional Independence Measure (FIM) [24,25]. The assessment consisted of 18 items, 13 of which were motor tasks and 5 were cognitive. Specifically, the FIM motor items include eating, grooming, bathing, dressing the upper body, dressing the lower body, toileting, bladder control, bowel control, transferring to bed/chair/wheelchair, transferring to toilet, transferring to tub/shower, walking or wheelchair use, and stairs. The FIM cognitive items are comprehension, expression, social interaction, problem-solving, and memory. Each item is scored on a scale of 1-7, and the total number of points is used to determine the final grade, which ranges from 18 to 126. The higher a person’s score, the more independent they are in performing daily activities.

The physical function, strength of the knee extensors, and ability to perform activities of daily living were assessed by the same investigator.

Assessment of sural nerve function

Sural nerve function was evaluated weekly from the time of admission using the DPNCheck (NeuroMetrix). Measurements were performed by the same investigator (E.T.) according to the manufacturer’s instructions. The measurements were performed at the patient's bedside with the patient in a comfortable supine position. After removing the horny layer of the skin at the test site, the stimulus probe was placed between the lateral malleolus and Achilles tendon, and CV and AP were measured using a biosensor located 9.22 cm away. Measurements were taken on the left and right lower limbs. The device was connected to a PC, and the readings were recorded each time using special software.

Statistical analysis

The Shapiro-Wilk test was used for the normality of demographic data, and the independent samples t-test or Mann-Whitney U test was used for the comparison of groups by sex. Fisher's exact test was used to compare categorical data. Effect sizes were calculated as Cohen's d for the independent samples t-test, r for the Mann-Whitney U test, and Cramer's v for Fisher's exact test. Pearson's product-moment correlation coefficient or Spearman's rank correlation coefficient was used to analyze the correlation between the sural nerve function and various measurement data, depending on the normality of the data. The average value measured during the hospitalization period was used as a representative value for the analysis of CV and AP. The average AP values during hospitalization were divided into two categories (greater than or less than 5 μV), and logistic regression analysis was performed using the data obtained at the time of hospitalization as the explanatory variable. Although clear criteria for peripheral neuropathy have not been established, the reason for setting the AP cutoff at 5 μV is that it is based on Baba's diabetic neuropathy classification [26], which is known as the severity classification for diabetic peripheral neuropathy. In addition, multiple regression analysis was conducted with the SPPB and FIM scores at the time of discharge as dependent variables, and the data collected at the time of admission and the average CV and AP during hospitalization as explanatory variables. The significance level was set at p < 0.05. Statistical analyses were performed using IBM SPSS Statistics for Windows, Version 29.0.2.0 (Released 2023; IBM Corp., Armonk, NY, USA).

## Results

**Figure 1:**
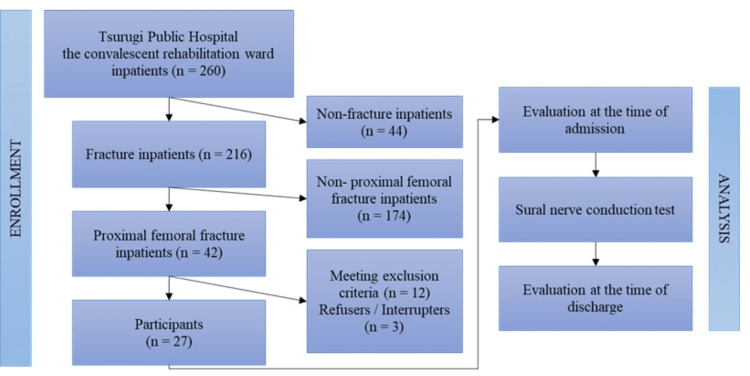
Flow chart depicting the study selection process.

During the study period, 42 patients with proximal femoral fractures were admitted to the hospital, 12 were excluded because of significant communication difficulties, and three refused to participate in the study. We enrolled 27 elderly patients with proximal femoral fractures (Figure 1).

Table 1 shows the results of the analysis of demographic characteristics classified by sex (male = 7, female = 20) and shows that males were significantly taller (165.9 ± 5.8 cm vs. 152.2 ± 5.9 cm, p < 0.001), and the CV of the sural nerve was significantly faster in females (43.5 ± 4.9 m/s vs. 49.2 ± 4.2 m/s, p = 0.006). Three patients experienced a fall while in the hospital, and all were female.

**Table 1:** Demographic characteristics classified by sex. Data are expressed as the mean with SD, median with IQR, or number and percentage of participants. Note: *p < 0.05; **p < 0.01; ^a^Fisher's exact test; ^b^Independent-samples t-test; ^c^Mann-Whitney U test Abbreviations: BMI, body mass index; CV, nerve conduction velocity; AP, action potential; MMT, manual muscle testing; SPPB, short physical performance battery; FIM, functional independence measure

Characteristics		Male (n = 7)	Female (n = 20)	p-values	Effect size
Age, years		82.9 ± 12.1	81.2 ± 5.8	0.731^b^	d = 0.219
Height, cm		165.9 ± 5.8	152.2 ± 5.9	<0.001^**b^	d = 2.334
Hospital admission BMI, kg/m^2^		20.4 ± 2.1	22.1 ± 3.0	0.189^b^	d = -0.593
Hospital discharge BMI, kg/m^2^		20.0 ± 2.2	21.7 ± 3.1	0.195^b^	d = -0.585
Waiting time for surgery, days (IQR)		7 (5-8)	5 (3-7)	0.464^c^	r = 0.150
Time from surgery, days (IQR)		19 (14-20)	19 (15-23)	0.935^c^	r = -0.016
Length of stay, days		41.1 ± 23.7	51.1 ± 21.4	0.314^b^	d = -0.451
Case of fall, n (%)		0 (0)	3 (15.0)	0.545^a^	V = 0.209
Medications at hospital admission					
Oral medication, type (IQR)		4 (1-4)	4 (1-7)	0.370^c^	r = -0.183
Nervous system drugs, n (%)		1 (14.3)	6 (30.0)	0.633^a^	V = 0.157
Cardiovascular system drugs, n (%)		2 (28.6)	7 (35.0)	1.000^a^	V = 0.060
Diabetes drugs, n (%)		0 (0)	4 (20.0)	0.545^a^	V = 0.247
Sural nerve function during hospitalization					
CV, m/s		43.5 ± 4.9	49.2 ± 4.2	0.006^**b^	d = -1.391
AP, μV (IQR)		5.1 (3.5-5.3)	5.3 (4.2-7.5)	0.400^c^	r = -0.171
MMT					
Hospital admission	Quadriceps R, grade (IQR)	5 (4-5)	4 (4-4)	0.048^*c^	r = 0.420
	Quadriceps L, grade (IQR)	4 (4-5)	4 (3-5)	0.219^c^	r = 0.259
Hospital discharge	Quadriceps R, grade (IQR)	5 (5-5)	5 (4-5)	0.240^c^	r = 0.272
	Quadriceps L, grade (IQR)	5 (5-5)	4 (4-5)	0.162^c^	r = 0.315
SPPB					
Hospital admission	Balance test, points (IQR)	4 (2-4)	4 (3-4)	0.935^c^	r = 0.018
	4-m walk test, points (IQR)	3 (2-3)	2 (1-2)	0.130^c^	r = 0.315
	Stand up test, points (IQR)	0 (0-1)	0 (0-1)	0.607^c^	r = -0.112
	Total, points	6.4 ± 2.0	5.9 ± 2.8	0.652^b^	d = 0.201
Hospital discharge	Balance test, points (IQR)	4 (4-4)	4 (3-4)	0.725^c^	r = 0.103
	4-m walk test, points (IQR)	3 (2-4)	3 (2-4)	0.850^c^	r = 0.039
	Stand up test, points (IQR)	1 (0-1)	2 (1-3)	0.219^c^	r = -0.247
	Total, points (IQR)	8 (5-9)	8 (7-11)	0.607^c^	r = -0.102
FIM					
Hospital admission	Total, points	79.6 ± 18.0	77.4 ± 14.7	0.747^b^	d = 0.143
	Motor items, points	49.4 ± 13.6	44.9 ± 12.1	0.411^b^	d = 0.367
	Cognitive items, points (IQR)	31 (25-34)	34 (31-34)	0.288^c^	r = -0.222
Hospital discharge	Total, points (IQR)	120 (99-122)	116 (110-120)	0.725^c^	r = 0.069
	Motor items, points (IQR)	87 (73-87)	82 (75-85)	0.533^c^	r = 0.123
	Cognitive items, points (IQR)	33 (26-34)	34 (30-35)	0.370^c^	r = -0.183

Table 2 shows the results of the correlation analysis between SPPB and FIM scores at the time of hospitalization and sural nerve function during hospitalization; a significant positive correlation was observed in several items. Among these, items with AP and |r| > 0.5 included balance on the SPPB (r = 0.520, p = 0.005), total FIM cognitive items (r = 0.575, p = 0.002), eating (r = 0.530, p = 0.005), toileting (r = 0.589, p = 0.001), bowel control (r = 0.524, p = 0.005), transfer to bed/chair/wheelchair (r = 0.521, p = 0.005), transfer to toilet (r = 0.535, p = 0.004), and problem-solving (r = 0.542, p = 0.004).

**Table 2:** Correlation analysis of SPPB, FIM, CV, and AP at hospital admission. Pearson's correlation coefficient or Spearman's rank correlation coefficient is reported. Note: *p < 0.05; **p < 0.01 Abbreviations: CV, nerve conduction velocity; AP, action potential; SPPB, short physical performance battery; FIM, functional independence measure

Variable	CV		AP	
	Correlation coefficient	p-value	Correlation coefficient	p-value
SPPB				
Balance test	0.238	0.232	0.520	0.005^**^
4-m walk test	-0.203	0.309	0.246	0.215
Stand up test	0.353	0.071	0.282	0.155
Total	0.057	0.777	0.406	0.036^*^
FIM				
Total FIM motor items	0.030	0.883	0.434	0.024^*^
Total FIM cognitive items	0.481	0.011^*^	0.575	0.002^**^
Self-care				
Eating	0.092	0.647	0.530	0.005^**^
Grooming	0.237	0.234	0.351	0.072
Bathing	-0.044	0.826	0.319	0.105
Dressing upper body	-	-	-	-
Dressing lower body	-	-	-	-
Toileting	-0.034	0.865	0.589	0.001^**^
Sphincter control				
Bladder control	0.034	0.867	0.343	0.080
Bowel control	0.150	0.454	0.524	0.005^**^
Transfers				
Bed/Chair/Wheelchair	0.067	0.741	0.521	0.005^**^
Toilet	0.076	0.705	0.535	0.004^**^
Tub/Shower	-0.227	0.255	-0.045	0.822
Locomotion				
Walking/Wheelchair use	-0.006	0.975	0.220	0.269
Stairs	-	-	-	-
Communication				
Comprehension	0.303	0.125	0.223	0.264
Expression	0.343	0.080	0.315	0.110
Social cognition				
Social interaction	0.182	0.365	0.418	0.030^*^
Problem solving	0.337	0.086	0.542	0.004^**^
Memory	0.341	0.082	0.479	0.012^*^

**Figure 2:**
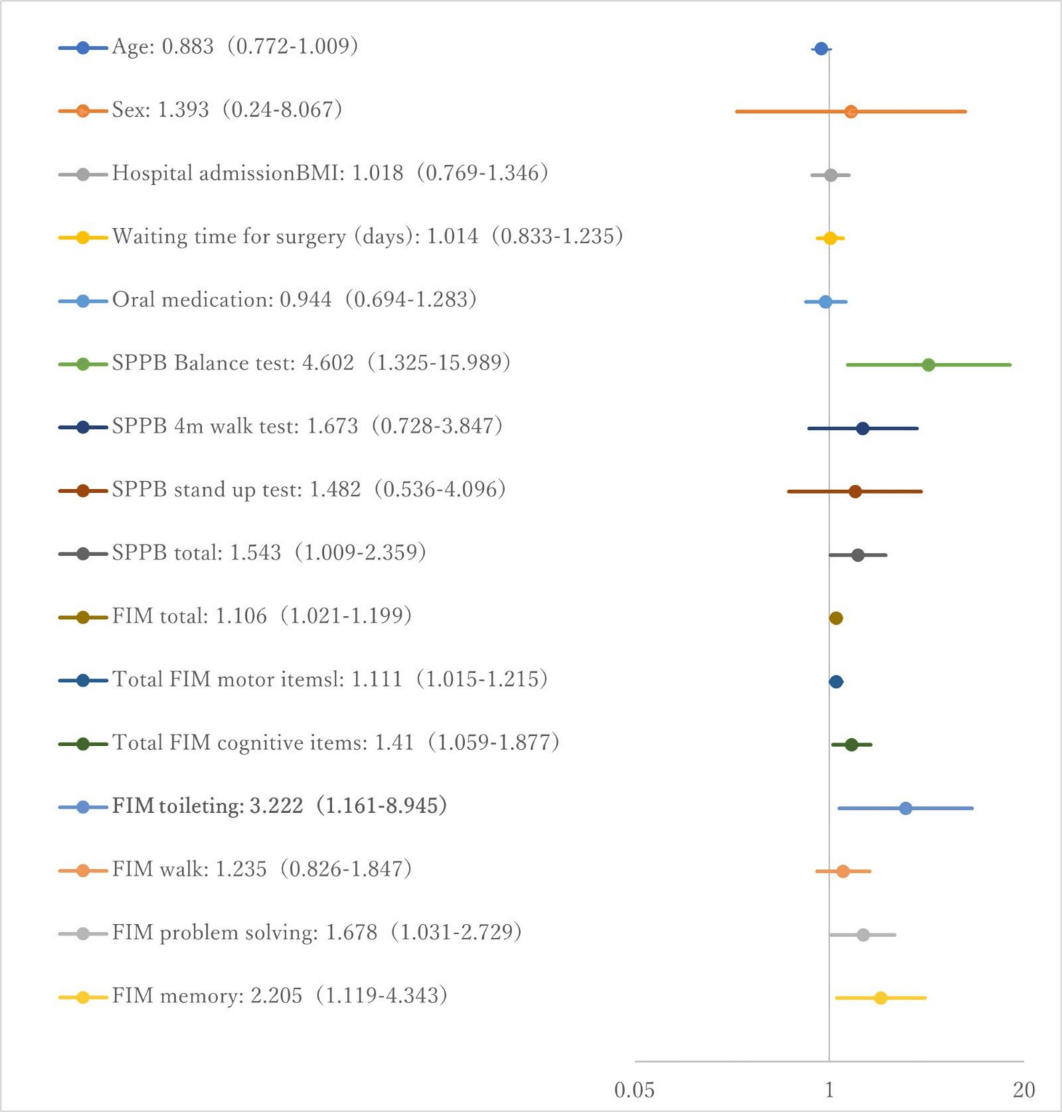
Univariate and multivariate analyses using binary logistic regression analysis, with AP <5 μV during hospitalization as the dependent variable and demographic data, and SPPB and FIM items as the explanatory variables. The figure shows odds ratios (95% confidence interval, lower limit, and upper limit) from the univariate analysis. Items in bold were selected as significant explanatory variables in the multivariate analysis. Abbreviations: AP, action potential; SPPB, short physical performance battery; MMT, manual muscle testing; FIM, functional independence measure; BMI, body mass index

Figure 2 shows the results of univariate and multivariate logistic regression analysis, where the dependent variable was the AP of the sural nerve during hospitalization (5 μV or more = 1/less than 5 μV = 0), and the explanatory variables were items suggested to be related to falls based on previous studies and data collected at the time of hospitalization [27-30]. In the multivariate analysis of the items used in the univariate analysis, based on the variance inflation factor (VIF) values, the two items with suspected multicollinearity - total FIM (VIF = 6.498) and total exercise items (VIF = 8.352) - were excluded, and all remaining items were included as explanatory variables. Variable selection was performed using the variable increment method based on the likelihood ratio, and FIM toileting was selected as a significant predictor in the final model (odds ratio: 3.22, 95% CI: 1.16-8.95, p = 0.025).

Table 3 shows the results of the correlation analysis between sural nerve function during hospitalization and SPPB and FIM scores at the time of discharge; a significant positive correlation was observed in several items. Among these, the items that showed a value of 0.5 or more with the AP were total FIM cognitive items (r = 0.609, p = 0.001), eating (r = 0.596, p = 0.001), bathing (r = 0.52, p = 0.005), toileting (r = 0.513, p = 0.006), problem-solving (r = 0.596, p = 0.001), and memory (r = 0.519, p = 0.006).

**Table 3:** Correlation analysis between CV and AP during hospitalization and SPPB and FIM at the time of discharge. Pearson's correlation coefficient or Spearman's rank correlation coefficient is reported. Note: *p < 0.05; **p < 0.01 Abbreviations: CV, nerve conduction velocity; AP, action potential; SPPB, short physical performance battery; FIM, functional independence measure

Variable	CV		AP	
	Correlation coefficient	p-value	Correlation coefficient	p-value
SPPB				
Balance test	0.026	0.897	0.391	0.044^*^
4-m walk test	0.095	0.638	0.467	0.014^*^
Stand up test	0.404	0.037^*^	0.429	0.025^*^
Total	0.257	0.195	0.465	0.014^*^
FIM				
Total FIM motor items	-0.013	0.949	0.452	0.018^*^
Total FIM cognitive items	0.446	0.020^*^	0.609	0.001^**^
Self-care				
Eating	0.121	0.547	0.596	0.001^**^
Grooming	0.098	0.625	0.496	0.009^**^
Bathing	0.063	0.755	0.528	0.005^**^
Dressing upper body	0.125	0.534	0.461	0.015^*^
Dressing lower body	0.004	0.983	0.461	0.015^*^
Toileting	0.035	0.862	0.513	0.006^**^
Sphincter control				
Bladder control	-0.208	0.298	0.295	0.135
Bowel control	0.034	0.865	0.398	0.040^*^
Transfers				
Bed/Chair/Wheelchair	0.013	0.947	0.273	0.168
Toilet	-0.025	0.901	0.221	0.268
Tub/Shower	0.111	0582	0.414	0.032^*^
Locomotion				
Walking/Wheelchair use	-0.128	0.523	0.236	0.235
Stairs	0.016	0.938	0.388	0.046^*^
Communication				
Comprehension	0.294	0.136	0.129	0.523
Expression	0.343	0.080	0.315	0.110
Social cognition				
Social interaction	0.182	0.365	0.418	0.030^*^
Problem solving	0.219	0.271	0.596	0.001^**^
Memory	0.326	0.097	0.519	0.006^**^

Table 4 shows the results of a multiple regression analysis in which the dependent variables were total SPPB, total FIM motor items, and total FIM cognitive items at the time of discharge. The explanatory variables were age, sex, BMI at admission, maximum grip strength at admission, number of medications at admission, MMT quadriceps strength (left and right), CV, and AP. A dummy variable was created for sex on a nominal scale. Observing the correlation matrix, there were no variables with |r| > 0.8. No items were suspected to be multicollinear according to the VIF. The results of the multiple regression analysis using the stepwise (variable increase/decrease) method showed that age was a significant explanatory variable for the total SPPB and FIM motor items. In contrast, the total FIM cognitive items included age and AP as significant explanatory variables, and the results of the analysis of variance (ANOVA) were significant, with an adjusted R² of 0.464. The Durbin-Watson ratio was 2.784, and there were no outliers where the predicted value was greater than ±3 SD of the actual measurement value.

**Table 4:** Multiple regression analysis was conducted with SPPB total, FIM motor total, and FIM cognitive total at discharge as dependent variables, and age, sex, BMI at admission, number of oral medications at admission, MMT quadriceps muscle strength (left and right), average CV during hospitalization, and AP as explanatory variables. Note: *p < 0.05; **p < 0.01 Abbreviations: CV, nerve conduction velocity; AP, action potential; SPPB, short physical performance battery; ANOVA, analysis of variance; FIM, functional independence measure; VIF, variance inflation factor

Hospital discharge	Non-standardization coefficient	Standardization coefficient	Significance probability	95% confidence interval	VIF	ANOVA		Multiple correlation coefficient	Coefficient of determination	Adjusted coefficient of determination
	B	B	p	Lower to upper	Value	p	F	R	R^2^	R^2^
SPPB total										
Constant	21.060	-	<0.001	9.625 to 32.495	-	0.027	5.517	0.425	0.181	0.148
Age	-0.159	-0.425	0.027^*^	-0.299 to -0.020	1.000					
FIM motor total										
Constant	132.017	-	<0.001	86.290 to 177.745	-	0.024	5.751	0.432	0.187	0.154
Age	-0.650	-0.432	0.024^*^	-1.208 to -0.092	1.000					
FIM cognitive total										
Constant	51.968	-	<0.001	36.978 to 66.958	-	<0.001	12.243	0.711	0.505	0.464
Age	-0.281	-0.525	0.002^**^	-0.449 to -0.112	1.129					
AP	0.489	0.333	0.039^*^	0.027 to 0.951	1.129					

## Discussion

In this study, the AP in sural nerve function was significantly associated with multiple items of the SPPB and FIM in elderly patients with proximal femoral fractures. Therefore, this study provides new evidence suggesting that AP in sural nerve function, which goes a step beyond previous research on lower extremity peripheral nerve disorders, may be a useful predictor of physical function and the ability to perform activities of daily living in elderly patients with proximal femoral fractures. As a result, it is possible that the early detection of a decline in physical and cognitive function through AP assessment in the early stages of rehabilitation intervention will contribute to the formulation of individualized fall prevention interventions related to the home environment during hospitalization.

Regarding the relationship between demographics, CV, and AP, the results showed that males were significantly taller than females and had a significantly slower CV. Previous studies have reported that CV is affected by height [31,32], and we believe that the same results were obtained in this study. The incidence of proximal femoral fractures in Japan is higher in women than in men [33,34], and the results of this study showed a higher proportion in women. This background suggests that it may have influenced the relationship between CV, SPPB, and FIM. Regarding AP, no differences were observed between sexes. In this study, falls during hospitalization were confirmed in 3 of the 27 patients (11.1%). Falls during hospitalization in patients with proximal femoral fractures in the last two years (April 2020 to March 2022) were confirmed in 23 of 96 patients (24.0%); we believe that we were able to extract a population with a lower risk of falls than in the past because of the exclusion criteria. In addition, the possibility that once-weekly bedside CV testing may play a role in fall prevention should be considered.

There was a significant correlation between SPPB and FIM scores at the time of hospitalization and CV and AP scores during hospitalization. In particular, there was a significant association between the FIM toileting score and the relationship between AP during hospitalization of less than 5 μV and physical function and mobility at the time of hospitalization. The association between falls and peripheral neuropathy has been reported in previous studies [18,35,36], and falls during hospitalization are often associated with toileting [37-39]. The results of this study suggest that a decrease in sural nerve function may be a factor in toilet-related falls during hospitalization. There are reports on the relationship between peripheral neuropathy, physical function, and motor ability [40], but we could not find any reports that focused on the CV and AP of the sural nerve, as in this study, and we believe that this is a novel finding. To prevent accidents related to toileting and defecation, incorporating an assessment of sural nerve function would be beneficial.

The CV and AP during hospitalization also showed a significant positive relationship with the SPPB and FIM scores at discharge. Similar to when the patient was hospitalized, AP showed a significant relationship with many items. In the multiple regression analysis, when the SPPB score at discharge and the total score of the FIM motor items were used as dependent variables, age was selected as a significant explanatory variable. However, when the total FIM cognitive item score was used as the dependent variable, age and AP were selected as significant explanatory variables. Peripheral neuropathy and cognitive decline have been previously reported [41], and we believe that the present study has shown similar results. The fact that these results were obtained even though people with severe cognitive decline were excluded from the study suggests that it is possible to detect mild cognitive impairment, and that the assessment of sural nerve function may be useful as an indicator of organic decline in brain function. There are reports that the presence or absence of cognitive decline and falls in hospitalized elderly patients is associated with an increased risk of falls, even when cognitive function is considered alone; however, this risk is further increased when mobility impairment and frailty are added to this condition [42-44]. The results of this study suggest that sural nerve AP is related to physical, motor, and cognitive function; therefore, as a single assessment index, it may play an important role in predicting falls.

The nerve conduction test, DPNCheck, used in this study is nearly painless for the patient and can be easily measured in a short time during routine medical examinations. Data obtained from this test suggest that its use in the general prediction of FIM, SPPB, and falls may be possible. In this study, measurements were taken every week, and the 27 participants were positive and cooperative.

This study had some limitations. First, the sample size is small. It is important to consider the possibility that the statistical power of the data from 27 individuals was low, and that selection bias and measurement error may have influenced the results. In the post-test power analysis of the correlation analysis in this study, when the significance level was set at 0.05 and the correlation coefficient was set at |r| = 0.5, the power was 80.6%, which is above the recommended power of 80% [45]. The results of this study should be interpreted with caution, but we believe that they have some explanatory power. Due to the small sample size, the statistical methods that can be used to analyze the data from this study are limited, and the results obtained must be interpreted with caution. The results obtained were interpreted according to the rules of the statistical analysis method used; however, it is believed that it will be necessary to increase the sample size in the future to improve the reliability of the results. Caution should also be exercised when generalizing the results, as this study only examined a single disease in a single hospital. However, if we combine all patients with fractures together, we will need to control for various confounding factors, such as fracture site, age, and mechanism of injury, and it is very likely that the results obtained will be difficult to interpret. The only lower limb peripheral nerve assessed was the sural nerve, and since the sural nerve is classified as a large-diameter fiber, caution must be taken in interpreting the results, as in previous studies, regarding the inability to assess small-diameter fiber peripheral nerve function [46]. However, the gold standard for the diagnosis of peripheral neuropathy is nerve conduction studies [47,48], and we believe that the fact that this study was able to relate CV and AP is a novel perspective.

In the future, the results obtained in this study will be useful for similar populations; however, it is necessary to expand the study to include research on fractures in different parts of the body and other diseases, as well as multicenter collaborative research to establish evidence. We also believe that it is important to conduct interventional studies and identify the factors that lead to improvements in peripheral nerve function.

## Conclusions

To our knowledge, this study is the first to demonstrate that sural nerve function may be a predictor of physical function and the ability to perform activities of daily living in patients with proximal femoral fractures. In particular, a significant association was observed between the sural nerve AP and several items of the SPPB and FIM at the time of admission and discharge. The results suggest that the assessment of sural nerve function may play an important role in rehabilitation planning aimed at managing fall risk and improving activities of daily living, and that more effective fall prevention interventions may be identified.
